# A type II implementation-effectiveness hybrid quasi-experimental pilot study of a clinical intervention to re-engage people living with HIV into care, ‘Lost & Found’: an implementation science protocol

**DOI:** 10.1186/s40814-020-0559-6

**Published:** 2020-02-21

**Authors:** Joseph Cox, Blake Linthwaite, Kim Engler, David Lessard, Bertrand Lebouché, Nadine Kronfli

**Affiliations:** 1 Chronic Viral Illness Service (CVIS), McGill University Health Centre (MUHC) – Glen Site, 1001, Decarie boulevard – D02.4110, Montreal, QC H4A 3J1 Canada; 2 Research Institute of the McGill University Health Centre (RI-MUHC), 2155 Guy Street, 5th Floor, Montreal, QC H3H 2R9 Canada; 3 Division of Infectious Diseases, Department of Medicine, Faculty of Medicine, McGill University, MUHC Glen Site Room E.05.1616, 1001 Boul. Decarie, Montreal, QC H4A 3J1 Canada; 4 Department of Epidemiology, Biostatistics, and Occupational Health, Faculty of Medicine, McGill University, Purvis Hall, 1020 Pine Avenue West, Montreal, QC H3A 1A2 Canada; 5 Department of Family Medicine, Faculty of Medicine, McGill University, 5858 Chemin de la Côte des Neiges, Montreal, QC H3S 1Z1 Canada

**Keywords:** Implementation science, Implementation strategies, Out-of-care, Lost to follow-up, HIV, Nursing, Re-engagement

## Abstract

**Background:**

At the McGill University Health Centre (MUHC), 10% of patients living with HIV do not return for care annually. Currently, no formal system exists to re-engage out-of-care (OOC) patients. Lost & Found, developed using an implementation science approach, is an intervention to re-engage OOC patients. It is based on existing evidence-based interventions and will be adapted for use by nurses at the MUHC. The aims of this study are to simultaneously assess both implementation and effectiveness of Lost & Found in order to determine the viability of a future multisite stepped-wedge cluster randomised trial.

**Methods:**

Lost & Found consists of two core elements: identifying and contacting OOC patients. Based on formative work involving MUHC nurses, and the use of a combined implementation framework (enhanced Replicating Effective Programs, Tailored Implementation for Chronic Diseases, and Proctor et al.’s implementation outcomes), we will adapt the intervention to our clinic. Adaptations include the creation of an OOC risk prediction tool, an automated real-time OOC list, and prioritization of high-risk OOC patients for re-engagement. Delivery and ongoing adaptation of the intervention will follow a three-pronged implementation strategy consisting of (1) promoting adaptability; (2) planning, engaging, executing, evaluating, and reflecting cycles; and (3) internal facilitation. This 15-month quasi-experimental pilot study adopts a type II implementation-effectiveness hybrid design. To evaluate implementation, a convergent parallel mixed-methods approach will guide the mixing of qualitative and quantitative data at time points throughout the study. In addition, descriptive and pre-post analyses, for each of the implementation and sustainability phases, will inform evaluations of the cumulative effectiveness and sustainability of the Lost & Found intervention.

**Discussion:**

This study will provide preliminary evidence for (1) the utility of our chosen implementation strategies and (2) the effectiveness of the intervention. Ultimately, this information may be used to inform future re-engagement efforts using implementation science in other HIV care centres. In addition, the procedures and measurement tools developed for this study will be foundational to the development of a multi-site, randomised stepped wedge study that would provide more robust evidence in support of the Lost & Found intervention.

## Background

The UNAIDS 90-90-90 targets have set ambitious and necessary global goals for improving the HIV care cascade and tackling the HIV epidemic by 2030 [1]. The latter two targets seek to ensure that 90% of people diagnosed with HIV are prescribed combination antiretroviral therapy (cART) and that 90% of these people have suppressed HIV viral loads (VL). To accomplish these objectives, people living with HIV (PLHIV) must be engaged in care.

The cascade of HIV care has been assessed at the McGill University Health Centre (MUHC) in Montreal, Quebec. Since 2015, 10% of registered patients have not returned for care annually [2]. Furthermore, a formal system to re-engage these patients does not exist. Given the individual- and population-level health impacts associated with suboptimal HIV treatment adherence, as well as the socioeconomic repercussions, attrition from the HIV care continuum must be minimised [3–5]. Therefore, developing and testing interventions to re-engage PLHIV into care should be prioritised.

Criteria used for defining and identifying patients as out-of-care (OOC) vary greatly. For example, definitions based on absenteeism differ on the time period selected, from a few months to greater than a year [3, 6–9]. Also, these definitions do not account for relevant clinical factors such as CD4 cell counts, viral load test results, and comorbidities, all of which may influence risks for negative HIV-related outcomes.

A definition for OOC that combines time- and patient-related characteristics could better inform patient-level HIV care trajectories. Using electronic medical record data on medication adherence, last care appointment, substance use, recent CD4 count, prior cART exposure, and treatment failure, Robbins et al*.* created a Risk Prediction Tool (RPT) to predict and stratify patients by risk of HIV viremia [10]. This RPT was later used to effectively predict patients’ risk of missing HIV care appointments [11].

Few interventions have been tested to re-engage PLHIV who are OOC. Upon identification and documentation of OOC patients—a necessary first step in re-engagement efforts—phone calls appear to be a simple and effective method for contacting and re-engaging OOC patients [3, 8, 9, 12]. Previous studies have used phone calls as part of a package of methods for contacting patients without evaluating their effectiveness alone [3, 8, 9, 12]. One of these studies attempted multiple phone calls (no more than three) over an undefined period of time [12]. Therefore, there appears to be evidence for using (1) clinical and administrative data to identify OOC patients and (2) phone calls to re-engage these patients into care.

In previous attempts to identify and re-engage OOC patients, MUHC nurses faced several barriers to implementation and sustainability, including competing priorities and limited resources (e.g. staffing shortages, technological limitations, and insufficient decision support). Among these re-engagement efforts was a manual, patient-by-patient assessment of the need for re-engagement using a paper list of all CVIS patients. Due to the abovementioned barriers, the nurses found it difficult to maintain this list and assure follow-up. Re-engaging OOC patients is considered within the scope of nursing practice and, despite a lack of formal or systematic efforts by nurses in the current model of care, their involvement in re-engagement efforts is likely to be key. Indeed, their clinical experience and knowledge about patients could be helpful in determining how and when patients should be re-engaged into care [13, 14].

## Lost & Found: an evidence- and stakeholder-informed intervention

Lost & Found is an intervention to re-engage OOC patients into HIV care. It was developed using an implementation science approach and will be implemented between 2018 and 2019 to improve the current standard of care. This intervention makes use of evidence-based interventions and adapts them for staff and the perceived needs of patients at the MUHC.

Adaptation of this intervention is promoted by differentiating its core elements from its adaptable peripheral components, consistent with processes of established implementation frameworks [15, 16]. Core elements are “the critical features of the design and intent of the intervention that are thought to be responsible for the intervention’s effectiveness” [15]. Peripheral components are aspects that can be flexibly adapted for different settings and that respond to observed challenges during delivery [15].

There are two core elements of the Lost & Found intervention: I. Identifying and documenting OOC patients and II. Contacting OOC patients. Within each core element, there are peripheral components which can be adapted during delivery. These are listed in Table 1.

**Table 1:** Peripheral components of each core element

I: Identifying and documenting OOC patients	II: Contacting OOC patients
1. OOC risk prediction tool 2. Automated, real-time list of OOC patients 3. Integration into the clinical EMR	1. Prioritization of higher risk patients 2. Nurse-led re-engagement 3. Nurse-delivered phone calls to OOC patients 4. Multiple contact attempts 5. Motivational communication

*OOC* = out-of-care; *EMR* = electronic medical record

### Core element I: Identifying and documenting OOC patients

**Fig. 1:**
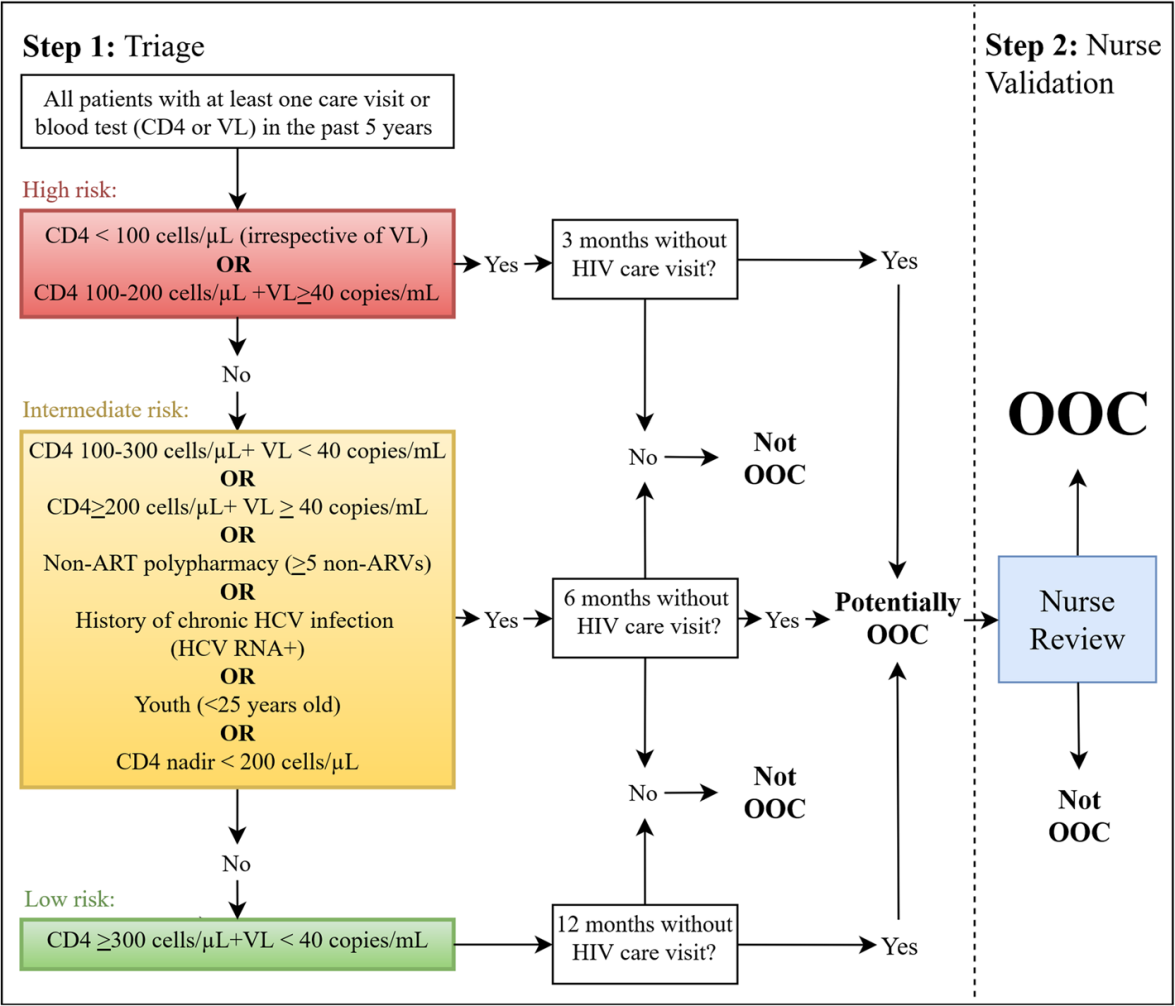
OOC risk prediction tool (OOC-RPT)

We developed a two-step OOC risk prediction tool (OOC-RPT) to identify OOC patients for care re-engagement (Fig. 1). The tool is based on evidence from the United States Department of Health and Human Services (DHHS) HIV treatment guidelines and was developed collaboratively with our primary Lost & Found stakeholders, MUHC nurses [17].

In step one of our OOC-RPT—“Triage”—all MUHC PLHIV are automatically classified daily as either high risk (red), intermediate risk (yellow), or low risk (green). These risk categories are informed by patients’ clinical characteristics. The OOC-RPT categories determine priority for re-engagement and are informed by the risk of disease progression. Patients are then classified as potentially OOC, based on time since their last appointment. Additional file 1 details the evidence supporting the use of these three categories and related criteria.

In step two—“Nurse Validation”—patients identified as potentially OOC are reviewed and confirmed as such by nurses. The need and urgency for re-engagement is determined based on risk category, information contained within the patient’s chart, and clinical judgement. Nurses’ knowledge of patients’ sociodemographic, psychosocial, and clinical factors, travel plans, shared care or other care arrangements, homelessness, mental illness, and/or chronic illness will help inform the confirmation of OOC status. All information related to follow-up of OOC patients will be documented in the clinic’s electronic medical record database, called RISQ (Réseau d’Informations Scientifiques du Québec).

The OOC-RPT will be programmed into RISQ to automate identification of OOC patients and provide a real-time list of OOC patients. Patients will be listed from high to low risk, helping nurses prioritize re-engagement efforts.

### Core element II: Contacting OOC patients

Clinic nurses will telephone patients using available contact information (RISQ, other hospital databases). They will receive a half-day of training from an expert in motivational communication, an essential skill for motivational interviewing. This training will help nurses encourage re-engagement through effective questioning, empathy, non-judgemental curiosity, guiding language, and adapted messaging [18]. This training is intended to provide nurses with additional skills or to strengthen existing skills for phone calls. It will be used at their discretion.

Patients who are reached by telephone will be scheduled for an appointment within timeframes dependent on their OOC-RPT risk category. However, earlier follow-up may be necessary as determined by clinical judgement. Patients in the high-risk category will be scheduled to be seen by a clinic nurse within 24-48 hours for basic primary care and phlebotomy (laboratory testing), and with their regular physician provider within one week of the nursing visit, for a full clinical assessment. Should the clinical situation suggest a need for more urgent care, the patient will be assessed by the walk-in physician on the same day as the nursing visit. Patients in the intermediate-risk category will be scheduled for an appointment within approximately two to four weeks of the telephone call, while patients in the low-risk category will be seen within four to six weeks. Patients who miss a scheduled appointment will be contacted the same day and provided another appointment, within timeframes depending on their risk category.

Attempts to contact patients will be made every 1, 2, and 4 weeks, for the high-, intermediate-, and low-risk categories, respectively. This pattern will be repeated until patients are contacted. Attempts to re-engage unreachable OOC patients will continue for up to 6 months (based on the maximum time a person can be outside of Quebec and still retain health care coverage), after which no further contact attempts will be made [19]. Nonetheless, these patients will remain on the OOC list until they return to care or until there is sufficient information to determine their care status. In all cases, nurses can modify the contact frequency and/or period based on individual patient factors.

## Proposed implementation of Lost & Found

We will use an implementation science approach to facilitate the adaptation and evaluation of Lost & Found in our clinic [16]. The findings from this study will highlight implementation strategies leading to successful local adaptation and real-world effectiveness [20].

### Implementation frameworks

Prior to selecting our implementation strategies, we developed a combined implementation framework for designing, implementing, and evaluating our clinical intervention and related implementation. It integrates elements of the following frameworks: enhanced Replicating Effective Programs (eREP) [21], Tailored Implementation for Chronic Diseases (TICD) [22], and Proctor et al.’s implementation outcomes [23]. eREP was used as the “process” framework to guide implementation of the intervention, TICD was utilised as the “determinants” framework to assist in understanding or explaining what might influence implementation, and Proctor et al.’s taxonomy of implementation outcomes was used as the “evaluation” framework [24]. Details regarding this combined implementation framework are outlined in Additional file 2. Based on formative work with MUHC nurses and the use of TICD worksheets [22], we identified 22 determinants (i.e. barriers and facilitators) that could impact the overall implementation of Lost & Found. We then categorised each determinant into the eREP phases (pre-implementation, implementation, or sustainability) based on when determinants were likely to have the greatest impact. Thereafter, this information was used to guide the selection of implementation strategies and related outcomes.

### Implementation strategies

**Fig. 2:**
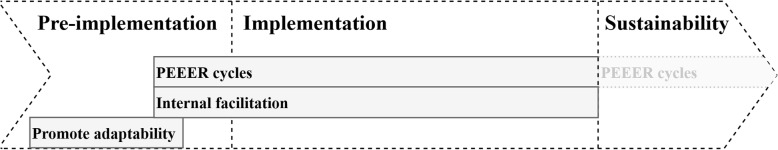
Implementation strategies by eREP implementation phase

We chose three core implementation strategies from available frameworks and inventories (eREP [21], TICD [22], and the Expert Recommendations for Implementing Change (ERIC) [25] project). These include (1) promoting adaptability; (2) planning, engaging, executing, evaluating, and reflecting (PEEER) cycles; and (3) internal facilitation. The times when these strategies will be used, relative to the eREP phases, are depicted in Fig. 2. Additional file 2 provides detailed descriptions of each implementation strategy and considers Proctor et al.’s guidelines for specifying and reporting implementation strategies [26].

#### Promote adaptability

During the pre-implementation phase, we customised and packaged the intervention into core elements and adaptable peripheral components (Table 1) [25]. The intervention, consisting of two core elements (i. identifying and documenting OOC patients, and ii. contacting OOC patients) and corresponding peripheral components, will be adapted to our setting over time. For example, the OOC-RPT (Fig. 1) and the role of nurses in re-engagement may change throughout implementation. This strategy helped prepare the intervention for adaptation and is an essential step in the eREP framework (Fig. 2) [21].

#### Planning, engaging, executing, evaluating, and reflecting cycles

We will make frequent changes to the peripheral components of the intervention through co-occurring cycles of planning, engaging, executing, evaluating, and reflecting (PEEER) initiated by nurses, the internal facilitator, or other research and clinical staff [16]. PEEER is the process through which adaptations to peripheral components of the intervention occur. This implementation strategy is often referred to as cyclical small tests of change [25]. PEEER may continue informally after implementation, leading to ongoing adaptations if needed.

#### Internal facilitation

The research coordinator, who has worked with the MUHC nurses on other projects, will serve as an internal facilitator and oversee the PEEER cycles. The internal facilitator will be available to nurses as needed, to address urgent barriers to implementation and to make time-sensitive adaptations when necessary. The study investigators will be consulted regularly and support the internal facilitator in engaging clinic stakeholders and ensuring timely adaptations. Regular and ongoing tasks carried out by the study team (investigators and internal facilitator) will be transferred to clinic staff at the end of the implementation phase/start of the sustainability phase.

## Research study aims

This quasi-experimental pilot study will follow a type II implementation-effectiveness hybrid design [27]. In type II implementation-effectiveness hybrid studies, the intervention and implementation strategy are given equal consideration and simultaneously evaluated. Alternatively, in type I and type III designs, the intervention and the implementation strategy, respectively, represent the primary focus for evaluation [27]. Fitting our need to both contribute to the evidence base on effectiveness for our intervention and test a combined implementation strategy, we chose a type II design. As a pilot study, we will learn whether the implementation of the intervention shows promise, possibly leading to a more rigorous evaluation using a randomised trial.

We envision implementing Lost & Found over twelve months followed by a sustainability phase of three months. This three-month period will provide time to document ongoing effectiveness and identify further changes that could help ensure long-term sustainability. All activities prior to implementation are considered part of the pre-implementation phase [21].

Under the overall objective of determining the viability of a multisite stepped-wedge cluster randomised trial, this study has two primary aims:

- Aim 1: To assess interim effectiveness and implementation outcomes of the Lost & Found intervention.
- Aim 2: To evaluate the cumulative effectiveness and short-term sustainability of the Lost & Found intervention.

## Methods

### Setting

The McGill University Health Centre (MUHC) is a large public quaternary care hospital in Montreal, Canada. Multidisciplinary care for adult patients with chronic viral illnesses such as HIV and chronic hepatitis C virus is provided at the Chronic Viral Illness Service (CVIS) clinic. Over 90% of patients who received care at the CVIS in 2018 were PLHIV (*n* = 1777). A variety of services are offered within the clinic including care by infectious disease and other specialists, family physicians, nurses, pharmacists, social workers, a psychologist, and a psychiatrist. The MUHC has a hospital-wide electronic medical record for all patients (OACIS) as well as one specifically designed for the CVIS (RISQ).

### Aim 1: To assess interim effectiveness and implementation outcomes

#### Overview

A convergent parallel mixed-methods approach will be used. Qualitative data on nursing-related implementation outcomes will be collected using focus groups, and a logbook maintained by the research coordinator/internal facilitator will document implementation and intervention changes throughout implementation. Quantitative data will also be collected using questionnaires to assess implementation outcomes and through RISQ, which will provide data on interim effectiveness outcomes. A mixed-methods analysis will allow for a more complete understanding of the impact of changes made to peripheral components of the intervention over time [28]. Implementation outcomes will help inform utility of the implementation strategy and serve as intermediate outcomes for interim effectiveness of the intervention, consistent with Proctor’s framework [23].

#### Participants

Participants will include all nurses providing clinical HIV care to patients over the study period (*n* = 4). For interim effectiveness outcomes, all PLHIV registered in the RISQ database during the 12-month implementation phase will be included. Patients who have not had an HIV care visit at the clinic within five years of the start of the study will be excluded.

#### Outcomes and related measures

##### Effectiveness

Information to evaluate changes in interim effectiveness outcomes over time will be collected automatically using RISQ and will be extracted for analysis at specific time points during the study (see Table 2). Interim effectiveness refers to effectiveness between the time points in Table 2, as opposed to cumulative effectiveness, which refers to overall effectiveness for the implementation or sustainability phases. These interim effectiveness outcomes include the number of patients identified and confirmed as OOC by nurses. Among OOC patients, we will report the number of patients contacted, successfully re-engaged, and not re-engaged, as well as reasons for lack of care re-engagement. For each OOC-RPT risk category, and overall, we will also provide the number of contact attempts needed to re-engage patients, time to re-engagement, and other clinical and sociodemographic information routinely collected in RISQ.

**Table 2:** Data collection schedule

	Pre-implementation	Week 1	Week 2	Week 3	Week 4	Month 2	Month 3	Month 6	Month 12	Month 15
Effectiveness					d	d	d	d	d	d
Feasibility	a, c				a, c	a, c	a, c	a, c	a, c	c
Acceptability	a, c				a, c	a, c	a, c	a, c	a, c	c
Adoption	a, c				a, c	a, c	a, c	a, c	a, c	c
Fidelity	b, c	b	b	b	b, c	b, c	b, c	b, c	b, c	c

a) 36-item questionnaire; b) fidelity checklist; c) focus group; and d) RISQ

##### Implementation

Four implementation outcomes will be studied (Additional file 3): (i) feasibility, i(i) acceptability, (iii) adoption, and (iv) fidelity.

Nurses will complete a 38-item self-administered questionnaire (Additional file 4) to assess implementation outcomes i to iii at various time points (see Table 2), each of which is captured by a 5-item scale. Scales for *feasibility and acceptability* were inspired by Weiner et al.’s pragmatic measures and the TAPP-C (The Arson Prevention Program for Children) compatibility and complexity subscales [29, 30]. These outcomes will be measured separately for each of the core intervention elements (i. identifying and documenting OOC patients, and ii. contacting OOC patients). *Adoption* will be measured using scales from the TAPP-C Adopter Characteristics Questionnaire and the TAPP-C Innovation Characteristics Questionnaire [29]. These questionnaires are comprised of subscales for factors that affect adoption: concern, self-efficacy, attitude, relative advantage, complexity, and compatibility. Given conceptual similarities to feasibility and acceptability, the complexity and compatibility subscales of the TAPP-C were not retained in the study questionnaire. Adoption subscales will focus on evaluating the combined intervention (core elements 1 and ii) so as to limit respondent burden. Justifications for the measures selected are provided in Additional file 3.

*Fidelity* will be assessed primarily through information automatically collected during the routine use of RISQ by nurses, and supplemented with simple checklists (Additional file 4). These self-administered checklists will assess fidelity to each core intervention element and peripheral components that cannot be measured through RISQ (for example, use of and adherence to motivational communication techniques). Nurses will also report on fidelity to motivational communication principles using questions inspired by the Behaviour Change Counselling Scale (BCCS) [18]. RISQ will provide all remaining fidelity information, such as how nurses use the OOC list.

Perceived *barriers and facilitators* to overall implementation, including the implementation outcomes of interest, will be assessed through seven focus groups with nurses. A timeline for these is presented in Table 2. These one-hour discussions will provide specific, actionable information for modifying peripheral components and contextualizing quantitative questionnaire data collected during the study. The focus groups from pre-implementation to month 12 will focus on factors related to implementation, while the focus group at month 15 will concentrate on sustainability of the Lost & Found intervention. Focus groups will be guided by a semi-structured interview schedule (Additional file 4), audio-recorded, and transcribed verbatim for a content analysis.

All modifications to peripheral components of the intervention will be documented in the internal facilitator’s logbook [31]. This will include an explanation of the change made, the actions taken to affect the change (i.e. the planning, engaging, and executing steps of the PEEER cycle), and the outcome of the change (i.e. the evaluating and reflecting steps of PEEER).

Nursing questionnaires will be administered and focus groups will be conducted more frequently during the first half of the project, where changes to peripheral components may be critical to overall implementation and effectiveness outcomes. The timing of data collection is summarised in Table 2 and is consistent with Proctor’s proposed salience of implementation outcomes relative to the implementation stage [23].

#### Data analysis

##### Quantitative analyses

To assess changes in interim effectiveness and nurse-related implementation outcomes throughout the study (Table 2), descriptive statistics (e.g. counts, proportions, medians, and inter-quartile ranges) will be reported.

All subscales for implementation outcomes will be scored using the mean of responses on individual 5-item Likert items. Subscale mean scores will be presented and a Cronbach's alpha will be calculated to report on the internal consistency of each subscale.

Throughout implementation, we will assess fidelity by reporting on the use of the OOC list and RISQ “follow-up” tab, as intended, as well as on adherence to each of the motivational communication principles. Other fidelity measures, such as the relative proportion of Lost & Found activities completed by each nurse (e.g. proportions of phone calls completed), will be drawn from the RISQ database. All analyses will be conducted using R statistical software.

##### Qualitative analyses

Focus group transcriptions will be analysed using a qualitative content analysis, concentrating on the manifest content of exchanges to provide a practical guide for action [32]. Deductive content analysis will be favoured, and barriers and facilitators identified by nurses will be placed into the relevant categories of TICD determinants or other novel, previously unidentified determinants. Fitting with the study’s longitudinal design, these determinants will be tracked over time [33]. Two qualitative data analysts will keep an audit trail of decision-making to allow for verification of the coding process and conclusions, with disagreements resolved by consensus. In addition to focus group data, each event in the facilitator’s logbook will be analysed using a realist analysis approach, assessing the context, mechanism, and outcome (CMO) of each documented change within the context of PEEER cycles. This approach is similar to the theoretical framework developed by Taylor et al. (2014) [34]. All qualitative data will be coded using the qualitative data management software, Atlas.ti version 8. The trustworthiness of the results will be verified by examining the analysts’ audit trails, presenting the results to nurses, and checking for consistency with the implementation outcomes.

##### Mixed-methods analyses

To guide the mixed-methods analyses, we will create a mixed-methods matrix whereby identified TICD determinants are mapped onto implementation outcomes. This was based on a causal chain developed to understand interrelationships between overall effectiveness and selected provider-related implementation outcomes (Additional file 3) [23]. This causal chain will also guide interpretation of our implementation outcomes.

After study completion, qualitative data and quantitative data will be presented together for each time point. Guided by the mixed-methods matrix, qualitative data will be used to provide nuance and context for the interpretation of quantitative data. Further context for nursing-related qualitative and quantitative results will be obtained from the qualitative analysis of changes documented in the internal facilitator’s logbook.

One of two results would suggest the viability of Lost & Found for trialing, ideally as a multisite stepped-wedge randomised study: (1) high and relatively stable overall scores for implementation outcomes throughout the study or (2) an overall increasing trend culminating in high end-of-study implementation outcome scores. Each of these would also be supported by qualitative data from focus groups with nurses.

### AIM 2: To evaluate cumulative effectiveness and sustainability

#### Overview

Three analyses will be undertaken:

i) A descriptive analysis of cumulative effectiveness during the implementation and sustainability phases;
ii) A pre-post evaluation of the proportion of OOC patients who are re-engaged during the implementation phase compared to the proportion of OOC patients in the year (2017–2018) prior to the implementation phase (2018–2019); and
iii) A pre-post evaluation of the proportion of OOC patients who are re-engaged during the sustainability phase compared to the last 3 months of the implementation phase.

#### Participants

All MUHC patients in the RISQ database during the 12-month implementation phase and the first three months of the sustainability phase will be included in analysis i). For analysis ii), we will retrospectively apply the OOC-RPT, excluding the nurse validation step, to patients in the year prior to implementation in order to create a pre-implementation comparison group. Thus, we will include all patients categorised as OOC by the OOC-RPT from one year prior (2017-2018) and to the end of the implementation phase (late 2019) in this analysis. Patients labelled as OOC before or after this time frame will receive the Lost & Found intervention, but will be excluded from the analysis because they did not become OOC within the time frame of the analysis. Based on the number of patients who were identified as not having had care appointments in 2016, we estimate approximately 25–30 patients will be identified as potentially OOC each month during the implementation of Lost & Found. In analysis iii), MUHC patients categorised as OOC from three months before the end of the implementation phase to the end of the sustainability phase (three months) will be included.

### Outcomes and related measures

#### Effectiveness

For i), the same effectiveness outcome measures detailed in Aim 1 will be used to report on cumulative effectiveness during the implementation phase (months 1 to 12) and for the sustainability phase (months 12 to 15) (Table 2). Re-engagement into care among OOC patients is the primary outcome of interest for ii) and iii).

#### Data analysis

i) Descriptive analysis

To assess cumulative effectiveness during implementation and sustainability, descriptive statistics (e.g. counts, proportions, medians, and inter-quartile ranges) will be reported.

ii) Pre-post analysis: Implementation phase

For this analysis, the second step of the OOC-RPT – nurse validation - will be ignored to ensure comparability between the two groups. This is done because nurse validation was not possible prior to the implementation of changes to the RISQ software. While this will result in a non-differential bias (to the null), it will simultaneously prevent a possible differential bias introduced from the nurse validation step of the OOC-RPT. To account for the impact that contact attempts could have on future follow-up behaviours, we will consider only the first OOC event for each patient. We will compare the re-engagement rates of OOC patients identified from our retrospective application of the OOC-RPT in the pre-implementation phase to re-engagement rates of OOC patients identified by step 1 of the OOC-RPT in the implementation phase. If the intervention is effective, we will find a statistically significant difference in the probability of re-engagement for OOC patients during the 12-month implementation phase compared to the year prior.

The impact of the complete Lost & Found intervention (i.e. both core elements) and implementation strategy on the proportion of OOC patients re-engaged will be examined in a Poisson model with robust variance estimation, using the year prior to implementation for comparison (see Eq. 1):

1 $$ \log \left(\mathrm{R}\right)={\upbeta}_1\ast \mathrm{Imp}+{\upbeta}_2\ast \mathrm{Sex}+{\upbeta}_3\ast \mathrm{Age}+{\upbeta}_4\ast \mathrm{Canada}+\upmu +\log \left(\mathrm{C}\right) $$

where *R* is a count of number of patients re-engaged into care, *Imp* is a dummy variable, where *Imp* = 1 indicates that the patient was OOC during the implementation phase and 0 for the pre-implementation phase, *Sex* accounts for the possible differences in the proportion of men and women in the pre-implementation or implementation phases, *Age* accounts for differences in the ages of patients between the two periods, and *Canada* accounts for differences between the two periods for the proportion of patients born outside of Canada, which may be of concern given a recent influx of refugees into our clinic. *log(C)* is considered the “offset” in the Poisson regression model, where *C* is the number of patients OOC over the two periods. We assume that Lost & Found will not impact the rate at which patients are categorised as OOC in the implementation phase and will verify this assumption prior to conducting the analysis. Potential other variables for inclusion in the model will be selected to adjust for possible temporal shifts in the patient population (i.e. factors that could affect engagement in care.) Due to lacking information in the pre-implementation period (e.g. number of patients confirmed as OOC by nurses, information about re-engagement efforts) it is not possible to consider the relative overall effectiveness of each core element.

The overall effect of the intervention and implementation strategy on the proportion of re-engaged OOC patients will be determined from the log of the *β*_*1*_ coefficient, providing a risk ratio for the probability of re-engaging OOC patients through the use of Lost & Found.

We did not conduct a sample size calculation due to uncertainty for several study parameters, including effect and sample sizes. The results of this analysis will inform power calculations for a larger study. Thus, while a statistically significant result would suggest that Lost & Found is viable for further evaluation, a statistically non-significant result would not necessarily rule it out since such a result may only be due to insufficient power.

iii) Pre-post analysis: Sustainability phase

This analysis is similar to the one detailed in ii) but compares effectiveness during the three-month sustainability phase to the last three months of the implementation phase. The analysis also differs in that the nurse validation step of the OOC-RPT will be included in each arm since manual changes to follow-up status is possible in both arms. We hypothesise that few changes will be made to peripheral components of Lost & Found in the last three months of the implementation phase, suggesting the intervention will be comparable to that which is delivered in the sustainability phase.

The same model (Equation 1) will be used to assess short-term sustainability of Lost & Found, where *Imp* = 1 indicates that the patient was OOC during the sustainability phase and 0 for the last three months of the implementation phase. A finding of no statistically significant difference in the probability of re-engagement for OOC patients in the implementation or sustainability phases will suggest short-term sustainability of Lost & Found.

All analyses will be conducted using R statistical software.

## Limitations

There are limitations to this pilot study. Firstly, our assessment of the implementation strategy is limited by our lack of a control group and our small sample (*n* = 4) of nurses. While our cumulative effectiveness and sustainability evaluations include control groups, pre-post analyses are subject to bias from uncontrolled confounding. Secondly, available clinical databases have insufficient information to accurately determine whether patients are truly OOC, which could inflate the number of patients identified as such. This is consistent with other studies finding that most patients identified as OOC are not actually OOC [35–37]. In our pre-post evaluations, this would have the effect of increasing the total number of OOC patients (the denominator) in each arm, biasing our effect estimates towards the null (i.e. non-differential bias). Finally, our assessment of sustainability is limited to three months, limiting our capacity to report on changes in Lost & Found use and related effectiveness over the long-term. Importantly, these limitations are offset by the variety of both intervention- and implementation-specific qualitative and quantitative assessments, as well as mixed-methods analyses. Together, we will have rich data for evaluating and reporting on the viability of implementing this intervention for the purposes of undertaking a larger scale and controlled evaluation.

## Conclusions

To our knowledge, this is the first study to address HIV care attrition using an implementation science approach. Our core intervention elements, consisting of identifying and contacting OOC patients, are simple enough to be easily adapted to other clinical settings. Additionally, our risk-based OOC definition is the first to incorporate patient characteristics thought to be associated with care interruptions.

Given the resource-intensive nature and low yield of previous efforts to identify and re-engage OOC patients [35–39], the capacity for adaptation in Lost & Found may help encourage uptake and optimise impact. Findings from assessments of the effectiveness of our complete intervention package and chosen implementation approach could inform similar efforts to re-engage PLHIV into care in other HIV care settings. If the combined intervention and implementation strategy are found to be successful, this study will provide the necessary information for carrying out a more robust evaluation of the effectiveness of the Lost & Found intervention and related implementation strategies.

### Trial status

Participant recruitment started in April 2018. Results of this study will be published.

## Supplementary information

**Additional file 1.** OOC-RPT_CoxLinthwaite

**Additional file 2.** Implementation_CoxLinthwaite

**Additional file 3.** OutcomesandInterrelationships

**Additional file 4.** DataCollectionTools_CoxLinthwaite

**Additional file 5.** DataManagement_CoxLinthwaite

## Data Availability

The data management procedures, study materials including questionnaires and implementation frameworks, and the datasets used and/or analysed during the current study are available from the corresponding author on reasonable request. Further detail can be found in Additional file 5. Results from this study will be shared with stakeholders at the MUHC and published in a peer-reviewed journal.

## Abbreviations

CVIS: Combination antiretroviral therapy
DSQ: Dossier santé Québec
EMR: Electronic medical record
HCV: Hepatitis C virus
HIV: Human immunodeficiency virus
MUHC: McGill University Health Centre
MSM: Men who have sex with men
OOC: Out of care, representing absenteeism from HIV care beyond what is expected for clinical status
PLHIV: People living with HIV
PWID: People who inject drugs
Re-engagement: Patients will be considered “re-engaged” in care if they re-present to the MUHC following a period of absenteeism
Retention: Patients will be considered “retained” in care if they remain in care one year following re-engagement
RISQ: Réseau d’informations scientifiques du Québec
VL: Viral load
