# Supplementary material for: A type II implementation-effectiveness hybrid quasi-experimental pilot study of a clinical intervention to re-engage people living with HIV into care, ‘Lost & Found’: an implementation science protocol
Source: Pilot Feasibility Stud. 2020 Feb 21;6:29. doi: 10.1186/s40814-020-0559-6 (PMC7035655; doi:10.1186/s40814-020-0559-6)
Supplement: Supplementary file 1 — Additional file 1. OOC-RPT_CoxLinthwaite [file 40814_2020_559_MOESM1_ESM.docx]

# **Additional file 1: OOC-RPT and integration into RISQ**

We developed a two-step OOC risk prediction tool (OOC-RPT) to identify OOC patients. The tool is based on evidence from the United States Department of Health and Human Services (DHHS) guidelines and was developed collaboratively with our primary Lost and Founds stakeholders, the MUHC nurses [18]. Using clinical characteristics and time since last visit, patients are categorized as high, intermediate and low risk for poor health outcomes. Using this information, the nurses then validate the patient’s OOC status and determine priority for re-engagement. This OOC-RPT is summarized in Figure 1.

**Figure 1**: OOC risk prediction tool (OOC-RPT)


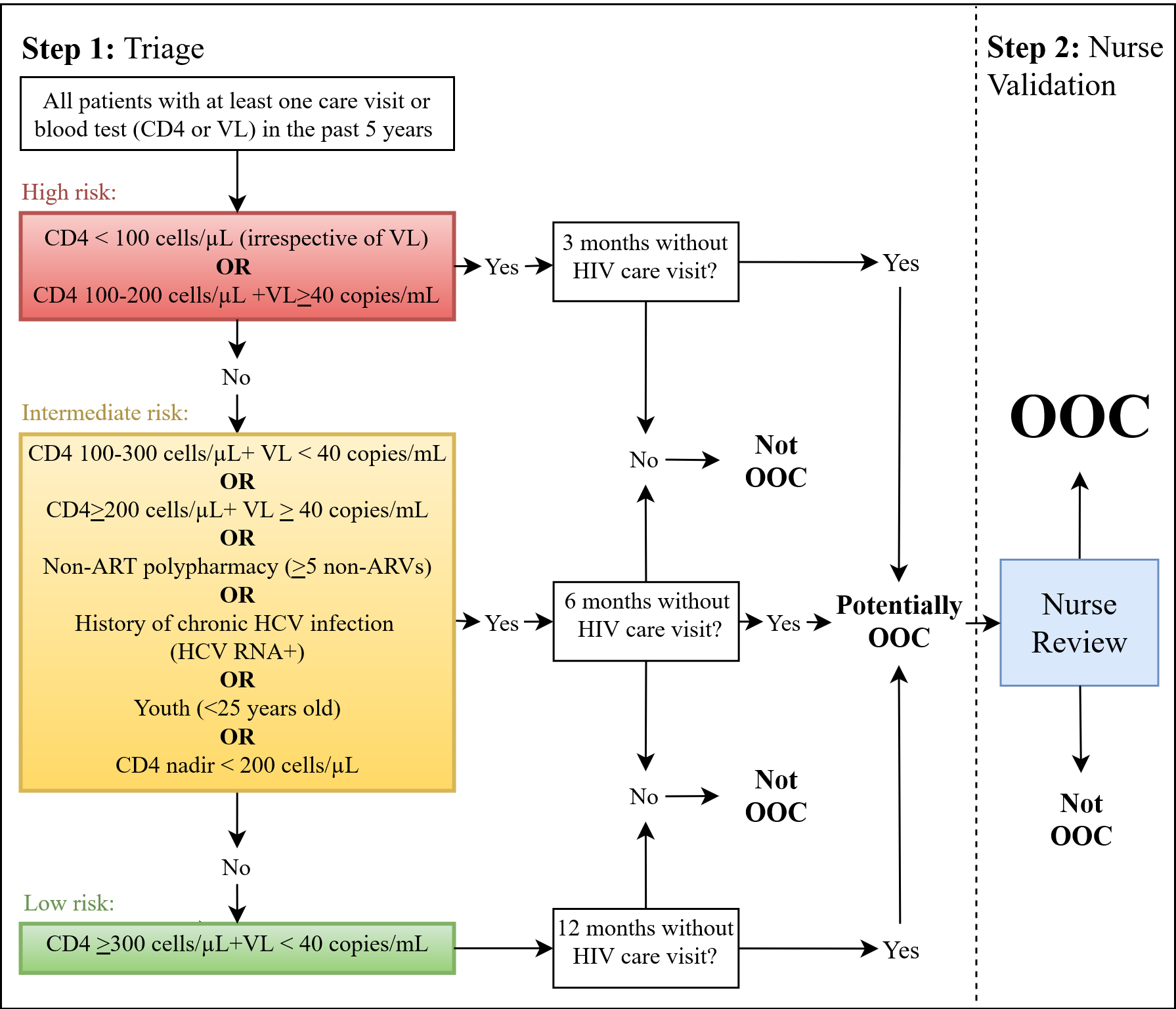


In step one of our OOC-RPT – “Triage” – all MUHC patients living with HIV and having had a clinic visit in the past five years are automatically classified daily into one of three risk categories: high risk (red), intermediate risk (yellow), and low risk (green). These risk categories are informed by patients’ clinical characteristics. Patients are then classified as potentially OOC based on their risk category and time since their last appointment. A five-year cut-off was applied for the OOC-RPT, decided by consensus with the primary investigators and nurses, based on the idea that people outside the five-year window are less likely to benefit from the intervention (i.e. have experienced other outcomes – moved, obtained care elsewhere, died, etc.).

The rationale for the OOC-RPT categories is informed by the risk of disease progression. A 12-month period of absenteeism is reasonable for well-controlled PLHIV [1]. However, this level of care would be considered inadequate for other patient types. For example, patients with a CD4 count < 200 cells/uL, are at higher risk for opportunistic infections and related hospitalizations, therefore, a three-month period of no care would be considered potentially OOC [1, 2]. PLHIV who have additional comorbidities such as the presence of non-antiretroviral polypharmacy (i.e., five or more non-HIV medications), will be considered OOC after a six-month period of no care as these patients typically require more regular follow-up (i.e., every 3-4 months) [3]. Several other factors constitute intermediate risk. For example, chronic hepatitis C virus (HCV) infection may accelerate the progression of HIV disease and may act as a proxy for other risk factors such as injection drug use [4, 5]. Thus, patients with a history of chronic HCV infection despite well-controlled HIV infection will be considered OOC after a six-month period of absenteeism as well. PLHIV under 25 years of age (i.e. youth) are less likely to be adherent to ART and more likely to experience viremia, necessitating more frequent follow-up [6-8]. CD4 nadirs of less than 200 cells/ul are predictive of larger HIV reservoirs and poor long-term HIV-disease outcomes [9, 10]. The lowest CD4 on record in the EMR will be used as a proxy for CD4 nadirs. Despite the availability of more sensitive tests, we use VL cut-offs of 40 copies/mL (or 20 copies/mL from November 2016). This is based on the usual practice of physicians and nurses, where repeat blood work to assess for potential viremia is done, even in the case of low-level virologic blips (defined as a detectable VL under 200 copies/mL after virologic suppression) [1].

In step two – “Nurse Validation” – patients potentially OOC are reviewed and validated by nurses. Based on clinical judgement, the need and urgency for re-engagement is determined. These decisions are informed by nurses’ knowledge of patients’ sociodemographic, psychosocial, and clinical factors, including access to care outside the MUHC, travel plans, shared care or other care arrangements, homelessness, mental illness, and/or chronic illness.

The OOC-RPT will be programmed into RISQ to 1) automate identification of OOC patients, and 2) provide a real-time list of OOC patients classified by OOC risk category (as determined by the OOC-RPT). The order of the list will help nurses prioritize re-engagement efforts, whereby patients in the high risk category will be placed at the top of the OOC list. This list will be displayed on the home page of the RISQ database, making it easily accessible to all clinical staff. In addition to information manually entered into RISQ, the program will automatically retrieve visit and clinical information from the hospital-wide EMR (OACIS), which will inform how patients are classified into the OOC risk categories. There will also be a new ‘follow-up’ section in RISQ, where nurses can: a) change patients’ risk categories at their discretion by entering information in the newly developed ‘follow-up’ section of RISQ, allowing for documentation of incarcerations, shared or informal care arrangements, deaths, and/or other reasons; and/or, b) validate patients’ OOC statuses and document information regarding re-engagement efforts. These changes will be automatically reflected in the OOC list. All changes in RISQ will be documented for the nurses’ reference, data collection, and to facilitate improvements to future iterations of the OOC-RPT.

References

1. US Department of Health. Guidelines for the use of antiretroviral agents in HIV-1-infected adults and adolescents. 2014.

2. US Department of Health. Guidelines for prevention and treatment of opportunistic infections in HIV-infected adults and adolescents. 2014.

3. Krentz HB, Gill MJ. The impact of non-antiretroviral polypharmacy on the continuity of antiretroviral therapy (ART) among HIV patients. AIDS patient care and STDs. 2016;30(1):11-7.

4. Bednasz CJ, Sawyer JR, Martinez A, Rose PG, Sithole SS, Hamilton HR, et al. Recent advances in management of the HIV/HCV coinfected patient. Future virology. 2015;10(8):981-97.

5. Chen F, Zhang J, Guo F, Wen B, Luo S, Yuan D, et al. HBV, HCV and HDV infection shows distinct patterns between injection drug users and general population. Journal of gastroenterology and hepatology. 2016.

6. Crepaz N, Tang T, Marks G, Mugavero MJ, Espinoza L, Hall HI. Durable viral suppression and transmission risk potential among persons with diagnosed HIV infection: United States, 2012–2013. Clinical Infectious Diseases. 2016;63(7):976-83.

7. O'connor JL, Gardner EM, Mannheimer SB, Lifson AR, Esser S, Telzak EE, et al. Factors associated with adherence amongst 5295 people receiving antiretroviral therapy as part of an international trial. The Journal of infectious diseases. 2012;208(1):40-9.

8. HIV/AIDS JUNPo, Organization WH. Young people and HIV/AIDS: opportunity in crisis. Young people and HIV/AIDS: opportunity in crisis. 2002.

9. Boulassel M-R, Chomont N, Pai NP, Gilmore N, Sékaly R-P, Routy J-P. CD4 T cell nadir independently predicts the magnitude of the HIV reservoir after prolonged suppressive antiretroviral therapy. Journal of Clinical Virology. 2012;53(1):29-32.

10. Buisker TR, Dufour M-SK, Myers JJ. Recall of Nadir CD4 Cell Count and Most Recent HIV Viral Load Among HIV-Infected, Socially Marginalized Adults. AIDS and Behavior. 2015;19(11):2108-16.
