# Supplementary material for: A type II implementation-effectiveness hybrid quasi-experimental pilot study of a clinical intervention to re-engage people living with HIV into care, ‘Lost & Found’: an implementation science protocol
Source: Pilot Feasibility Stud. 2020 Feb 21;6:29. doi: 10.1186/s40814-020-0559-6 (PMC7035655; doi:10.1186/s40814-020-0559-6)
Supplement: Supplementary file 2 — Additional file 2. Implementation_CoxLinthwaite [file 40814_2020_559_MOESM2_ESM.docx]

# **Additional file 2: Implementation of Lost & Found**

**Table of Contents**

[i) Formative work 2](#_Toc11157886)

[ii) Combined Implementation Framework 3](#_Toc11157887)

[iii) Implementation strategy and evaluation plan development 3](#_Toc11157888)

[iv) Combined implementation strategy 6](#_Toc11157889)

## **Formative work**

The Lost & Found intervention was first conceived following an analysis of the 2016 HIV care cascade for the MUHC. We found that 10% of patients with visits in 2015 did not return for care. A similar proportion of patients did not return for care in 2016. This pattern was concerning given the known negative individual and societal health and economic costs associated with HIV care attrition. The earliest conception of the intervention entailed a list of patients without any care visits in the past year, printed monthly for nurses to review, triage, and contact each patient as necessary.

In a meeting between some of the nurses and the investigative team, the nurses questioned the utility of a list of patients with year-long care absences. Specifically, they felt that some patients with severe HIV-related illness or poorly managed co-morbidities could be considered out of care (OOC) much earlier than at one year. In a follow-up meeting with nurses, the research coordinator attempted to summarize their logic in a systematic manner, consisting of three different groups of people who are OOC after three, six, or twelve months, depending on their risk of negative HIV or other health outcomes. Over the course of regular, mostly informal meetings with the research coordinator acting as a liaison between the nurses and study investigators, and two formal meetings between the entire team, an OOC risk prediction tool (OOC-RPT; Additional file 1) was developed based on original efforts by the MUHC nurses, their perception of patients’ needs, and DHHS guidelines, while also considering data limitations at the MUHC [1]. These consultations between the study team, the MUHC nurses and other stakeholders (e.g., nurse manager, pharmacist representative) over the course of several months allowed the study team to develop an understanding of determinants, positive or negative, that could impact successful implementation, including those related to the intervention itself, the MUHC team, and the clinic.

## **Combined Implementation Framework**

We combined elements of the Enhanced Replicating Effective Programs framework (eREP), the Tailored Implementation for Chronic Diseases framework (TICD), and Proctor et al.’s implementation outcomes framework into a single framework for designing, implementing, and evaluating clinical interventions and implementation strategies, depicted in Figure A1 [2-4]. This framework adopts the eREP timeline for different implementation phases and builds upon the taxonomy of implementation outcomes established by Proctor et al., by adding barriers and facilitators from the TICD checklist and corresponding implementation strategies as integral to success on implementation outcomes. This combined implementation framework is conceptualized from top to bottom in our diagram, and was used to guide the determination of implementation strategies and the evaluation plan. The final stage of eREP, ‘Dissemination,’ was renamed ‘Sustainability’ to better reflect the needs of single-site clinical interventions.

## **Implementation strategy and evaluation plan development**

TICD worksheets one, two, and three were completed in order to identify important implementation barriers and facilitators, and to help develop a corresponding implementation strategy [3].

In TICD Worksheet One, three investigators (JC, NK, BL) independently identified the specific recommendations that constitute Lost & Found and assessed the potential impact of non-adherence to these recommendations on successful implementation. Discrepancies were addressed by consensus. In total, 10 recommendations were identified. Five out of 10 recommendations were assessed in subsequent worksheets because they were scored above our chosen cut-off score of four out of five points for the question “is implementing the recommendation a priority?”: 1) Generation of the OOC list; 2) nurse validation and use of the OOC list; 3) nurse phone calls to patients; 4) regular input of patient information into the RISQ follow-up tab; and 5) repeated phone calls to uncontacted OOC patients.

**Figure A1:** Combined implementation framework


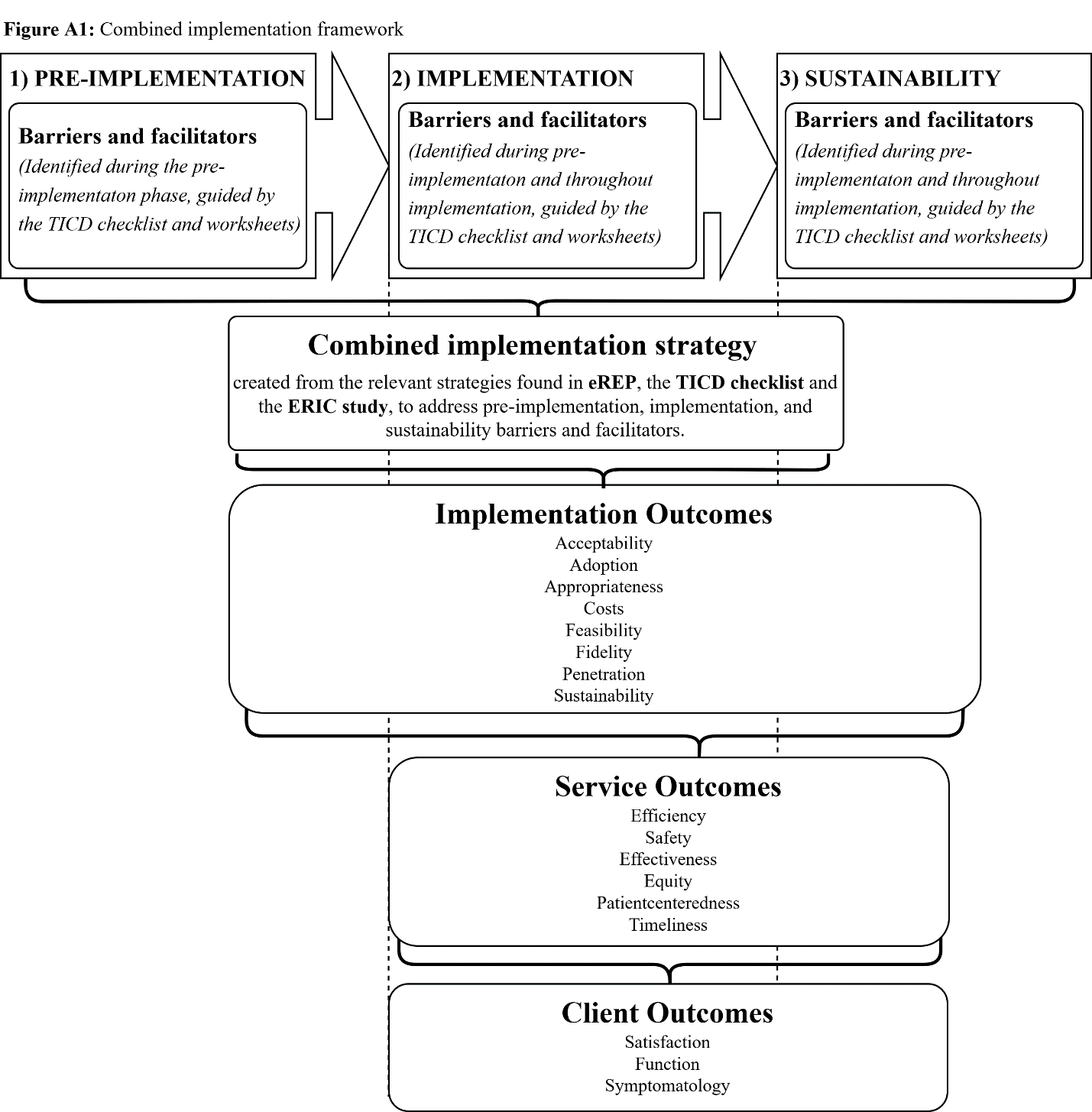


In TICD Worksheet Two, all three investigators (JC, NK, BL) assessed the potential for impact (either positive or negative) of every determinant in the TICD checklist on the five recommendations. 33/57 determinants were identified as potentially impactful on at least one of the recommendations. For example, we identified the amount of effort necessary to implementation Lost & Found as a barrier and the trialability of the intervention as a facilitator. The reasons for their suspected impact were documented in the worksheet, along with next steps for investigation.

In TICD Worksheet Three we documented the likely impact of each of the 33 determinants on successful implementation, evidence for their impact, an impact score for the determinant ranging from negative three to three, and possible implementation strategies to respond to these determinants. Possible implementation strategies were drawn from the TICD checklist, eREP, and the Expert Recommendations for Implementing Change (ERIC) project [2, 3, 5]. This worksheet was completed by one investigator (BL) whose work was reviewed individually by two others (JC, NK). Discrepancies were solved by consensus. In total, 22 of 33 determinants were given an impact score with a magnitude of two or greater out of three (i.e. they could significantly impact implementation) and were included in the final step to develop a combined implementation strategy.

To develop the combined implementation strategy for Lost & Found, rather than use TICD Worksheet Four, the 22 barriers and facilitators were organized into categories by eREP implementation phase. One barrier or facilitator could be placed under multiple categories if it was considered impactful at multiple stages. For example, ‘availability of necessary resources’ is listed under each of the phases as an example in the framework for our diagram (Figure A1). This list of barriers and facilitators for each stage of implementation was used as a checklist of determinants to be addressed on by the combined implementation strategy. Guided by this checklist and the possible implementation strategies identified in TICD Worksheet Three, we developed a combined implementation strategy adapted to the needs of the CVIS. We identified nine strategies that could be useful for addressing barriers and optimize upon facilitators for implementing Lost & Found.

For barriers and facilitators in the ***pre-implementation phase***, we identified:

1. Engage stakeholders;
2. Customize appropriate intervention; and
3. Promote adaptability.

For barriers and facilitators in the ***implementation phase***, we identified:

1. Training and orientation;
2. Internal facilitation;
3. Task shifting;
4. Planning, engaging, executing, evaluating, and reflecting cycles; and
5. Ongoing stakeholder engagement.

For barriers and facilitators in the ***sustainability phase***, we identified:

1. Integrate recommendations and tools into routine care;
2. Reduce the need for additional resources; and
3. Evaluate and disseminate outcomes.

These nine were condensed into a ***combined implementation strategy*** consisting of three core implementation strategies that allowed for the broadest coverage in addressing the determinants identified for each implementation phase:

1. Promote adaptability;
2. Planning, engaging, executing, evaluating, and reflecting cycles; and
3. Internal facilitation.

We hypothesize that this combined implementation strategy will act as a positive mediator for the effects of barriers and facilitators, and the implementation outcomes relevant to our study. In this way, our combined implementation strategy is akin to an intervention in traditional effectiveness research; however, the primary outcomes of the implementation strategy are related to implementation rather than effectiveness [6]. Outcomes were selected for evaluation in our study because they were most directly related to or impacted by the primary implementation stakeholders, MUHC nurses. The other implementation outcomes were deemed less relevant for successful implementation of Lost & Found at the CVIS based on the determinants identified, and thus, not included in our evaluation plan.

## **Combined implementation strategy**

We chose three core implementation strategies from eREP, TICD, and the Expert Recommendations for Implementing Change (ERIC) project that could be useful for implementing Lost & Found [2, 3, 5]. These strategies were selected to address identified determinants during implementation: 1) promoting adaptability; 2) planning, engaging, executing, evaluating, and reflecting (PEEER) cycles; and 3) internal facilitation. The timing of these strategies relative to the eREP continuum is depicted in Figure 2. Each implementation strategy is described below considering the dimensions for specifying and reporting implementation strategies by Proctor et al.[6].

### **1) Promote adaptability**

Early in the pre-implementation phase, study investigators customized and packaged the intervention into core elements and peripheral components. This was a necessary first step to determine which parts of the intervention can be adapted (i.e. the peripheral elements), while maintaining the core elements of the intervention. This is one of the essential steps in the eREP framework [2]. The aspects of the intervention which fall into core or peripheral can change in collaboration with stakeholders, as represented by the overlap with the PEEER cycles and internal facilitation strategies.

#### **2) Planning, engaging, executing, evaluating, and reflecting cycles**

We made and will continue to make frequent changes to the peripheral elements of the intervention through cycles of planning, engaging, executing, evaluating, and reflecting (PEEER) [7]. This process is analogous to the plan-do-study-act (PDSA) cycle in quality improvement efforts, and referred to as ‘cyclical small tests of change’ in ERIC [8]. However, we will adhere more closely to the language used by the Consolidated Framework for Implementation Research (CFIR) [7]. We conceptualize this “cycle of change” in terms of five steps: 1) planning; 2) engaging 3) executing; 4) evaluating; and 5) reflecting. This process began after the intervention was packaged into core and peripheral elements.

*Planning* refers to the development of schemes or methods to adapt the intervention or its implementation [7]. These plans could be ‘top-down,’ meaning they are initiated by the study investigators, ‘bottom-up,’ meaning they are initiated by intervention stakeholders (nurses), or ‘shared,’ meaning they are initiated by both nurses and study investigators after group deliberations.

*Engaging* refers to attracting and involving important stakeholders, who can propel or derail change efforts [7]. Engagement of clinicians and other critical decision makers is an important step for overcoming barriers to implementation in the health care system [9]. Given that Lost & Found is primarily a nurse-led intervention, nursing engagement in the intervention and its implementation is central to success. In fact, this project builds upon nurses’ previous efforts to re-engage OOC patients. Nurses have already provided input for the majority of project components, including the OOC-RPT and the changes to RISQ. Input from an HIV patient advisory group will also be sought to refine project tools and protocols. In addition to engaging nursing and patient stakeholders, we have reached out to administrators, receptionists, and pharmacists, all of whom have been previously implicated in re-engaging OOC patients at the MUHC or could potentially impact the implementation of Lost & Found.

*Executing* refers to the concrete actions taken to make these adaptations [7]. Depending on the nature of the action, this will be carried out by the research coordinator, the nurses, or the primary investigators. For example, changes to RISQ would be carried out by the research coordinator in collaboration with a software programmer, while nurse-specific adaptations, such as task shifting to manage multiple responsibilities, would be carried out primarily by the nurses themselves.

*Evaluating* refers to qualitative or quantitative feedback about the specific adaptation, or the intervention and implementation as a whole [7]. Nurses will receive this feedback through RISQ and the OOC list. The results of every analysis, throughout or after implementation, will be disseminated to nurses.

*Reflecting* refers to contemplation about the qualitative or quantitative results and their implications [7]. This final step of the cycle is expected to occur independently among nurses, or when debriefing with the research coordinator informally throughout implementation or more formally during focus groups. This final reflection step informs the planning step of the next cycle of change. Where important barriers or sub-optimal implementation occurs, the research coordinator will discuss/communicate with the investigative team so as to implement potential changes to peripheral elements of the intervention or combined implementation strategy. Based on feedback received from nurses, changes to the peripheral elements of the intervention and implementation strategy will be made within a maximum of two weeks.

These steps of the PEEER cycle do not need to occur in order and could start at any point in the cycle. For example, while executing change, it may become apparent that the necessary stakeholders have not been engaged, at which point efforts would be made to do so. These changes will occur frequently throughout the pre-implementation and implementation phases, often simultaneously for different aspects of the intervention. For example, several cycles of changes to RISQ could occur while one cycle of change is occurring for the content of phone calls. While formal PEEER cycles will not continue after the implementation phase, PEEER could continue informally when adaptations are needed, if initiated by clinical stakeholders, without the support of this study, and using clinical resources.

#### ***3) Internal facilitation***

Facilitation is a systematic and iterative process in which implementation experts promote program uptake by working with frontline providers to identify and mitigate barriers to program adoption, and is an effective means for ensuring intervention uptake [2]. Internal facilitation is facilitation performed by someone from within the setting, as opposed to external facilitation, which is done by an outside party [10]. The research coordinator, who has worked with the MUHC nurses on other projects and has established a professional relationship, will serve as an internal facilitator and oversee the PEEER cycles. In addition to regular focus groups with nurses to identify barriers and facilitators, the research coordinator will be regularly available to MUHC nurses during implementation to address urgent barriers to implementation and to make time-sensitive changes where necessary.

References

1. US Department of Health. Guidelines for the use of antiretroviral agents in HIV-1-infected adults and adolescents. 2014.

2. Kilbourne AM, Abraham KM, Goodrich DE, Bowersox NW, Almirall D, Lai Z, et al. Cluster randomized adaptive implementation trial comparing a standard versus enhanced implementation intervention to improve uptake of an effective re-engagement program for patients with serious mental illness. Implementation Science. 2013;8(1):136.

3. Flottorp SA, Oxman AD, Krause J, Musila NR, Wensing M, Godycki-Cwirko M, et al. A checklist for identifying determinants of practice: a systematic review and synthesis of frameworks and taxonomies of factors that prevent or enable improvements in healthcare professional practice. Implementation Science. 2013;8(1):35.

4. Nilsen P. Making sense of implementation theories, models and frameworks. Implementation Science. 2015;10(1):53.

5. Powell BJ, Waltz TJ, Chinman MJ, Damschroder LJ, Smith JL, Matthieu MM, et al. A refined compilation of implementation strategies: results from the Expert Recommendations for Implementing Change (ERIC) project. Implementation Science. 2015;10(1):21.

6. Proctor EK, Powell BJ, McMillen JC. Implementation strategies: recommendations for specifying and reporting. Implementation Science. 2013;8(1):139.

7. Damschroder LJ, Aron DC, Keith RE, Kirsh SR, Alexander JA, Lowery JC. Fostering implementation of health services research findings into practice: a consolidated framework for advancing implementation science. Implementation science. 2009;4(1):50.

8. Institute for Healthcare Improvement. The Breakthrough Series: IHI's Collaborative Model for Achieving Breakthrough Improvement. Innovation Series. 2003.

9. Concannon TW, Fuster M, Saunders T, Patel K, Wong JB, Leslie LK, et al. A systematic review of stakeholder engagement in comparative effectiveness and patient-centered outcomes research. Journal of general internal medicine. 2014;29(12):1692-701.

10. McCullough MB, Gillespie C, Petrakis BA, Jones EA, Park AM, Lukas CV, et al. Forming and activating an internal facilitation group for successful implementation: A qualitative study. Research in Social and Administrative Pharmacy. 2017.
