# Supplementary material for: A type II implementation-effectiveness hybrid quasi-experimental pilot study of a clinical intervention to re-engage people living with HIV into care, ‘Lost & Found’: an implementation science protocol
Source: Pilot Feasibility Stud. 2020 Feb 21;6:29. doi: 10.1186/s40814-020-0559-6 (PMC7035655; doi:10.1186/s40814-020-0559-6)
Supplement: Supplementary file 3 — Additional file 3. OutcomesandInterrelationships [file 40814_2020_559_MOESM3_ESM.docx]

# **Additional file 3: Selected outcomes and interrelationships**

## **Outcome definitions:**

***Acceptability*** is the is the perception among implementation stakeholders that a given treatment, service, practice, or innovation is agreeable, palatable, or satisfactory [1].

***Adoption*** is the intention, initial decision, or action to try or employ an innovation or evidence-based practice. It is also referred to as ‘uptake’ [1].

***Feasibility*** is the extent to which a new treatment or innovation can be successfully used or carried out within a given agency or setting [1].

***Fidelity*** is the degree to which an intervention was implemented as it was prescribed in the original protocol or as it was intended by the program developers [1]. There are five elements of fidelity: adherence to an intervention; exposure or dose; quality of delivery; participant responsiveness; and program differentiation [2].

***Effectiveness*** is the real-world impact of the intervention.

Appropriateness (defined as the perceived fit, relevance, or compatibility of an innovation or evidence based practice for a given practice setting or to address a particular problem) is conceptually similar to acceptability, but is generally measured as a distinct outcome [1]. However, for this study we consider them as complementary concepts and have incorporated measures of appropriateness into our acceptability measures, where applicable.

## **Interrelationships between selected provider-related outcomes**

To guide our mixed-methods analyses and interpretation of implementation outcomes, we developed a causal chain for the interrelationships between our selected provider-related outcomes, inspired by Proctor et al. and summarized in Figure A2 [1].

**Figure A2:** Causal chain of provider-related outcomes


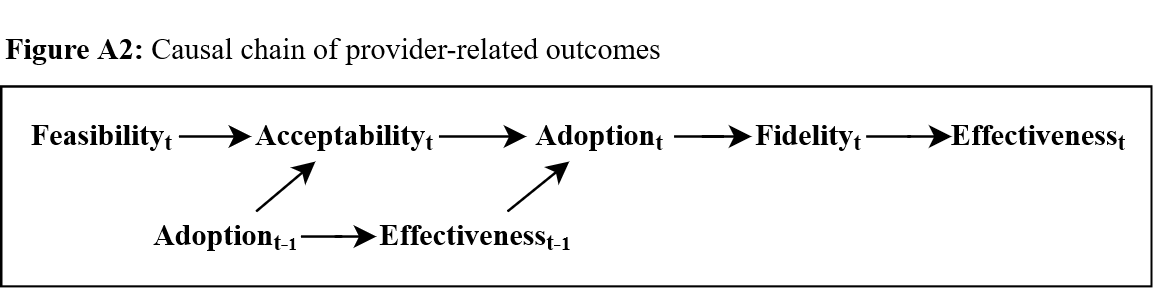


Providers’ perception of the feasibility of an intervention at time $t$ will likely impact their acceptability of the intervention [1]. Intervention acceptability will likely affect the decision to adopt an intervention [1]. This decision will influence fidelity to the intervention, which will ultimately impact effectiveness of the intervention as a whole [1, 2]. However, consistent with Rogers’ theory of the diffusion of innovation, the ability to adopt or adapt an innovation may increase its acceptability [1, 3]. Thus, ${Adoption}_{t-1}$ represents either a previous attempt to adopt the intervention or a preconceived notion they have about their ability to adopt the intervention. ${Effectiveness}_{t-1}$ represents the effectiveness of the intervention at earlier time points *throughout implementation*. Therefore, if t=1, the ${Effectiveness}_{t-1}$ term would be non-existent. For t>1, ${Effectiveness}_{t-1}$ influences the perceived relative advantage of the intervention (a subscale of adoption) at time $t$ [4]. For example, if an intervention were perceived as less effective than standard of care, both relative adoption and fidelity to the intervention would decrease, which would reduce the overall effectiveness.

In questionnaires administered to nurses (Additional file 4), we will measure acceptability, feasibility, and fidelity for each of the intervention’s core elements separately, but will measure adoption for the combination of core elements; this is done to reduce response burden. We used this causal chain to justify this choice; for a given core element, we believe we will be able to identify sub-optimal adoption based on low levels of acceptability. For example, if the phone calls are not accepted, we will find lower levels of overall adoption, and likely lower levels of fidelity to the phone calls. Qualitative data from focus groups will provide additional context.

This model will be used to guide the development of a mixed-methods matrix, whereby TICD determinants will be mapped onto our selected nurse-related implementation outcomes.

References

1. Proctor E, Silmere H, Raghavan R, Hovmand P, Aarons G, Bunger A, et al. Outcomes for implementation research: conceptual distinctions, measurement challenges, and research agenda. Administration and Policy in Mental Health and Mental Health Services Research. 2011;38(2):65-76.

2. Carroll C, Patterson M, Wood S, Booth A, Rick J, Balain S. A conceptual framework for implementation fidelity. Implementation science. 2007;2(1):40.

3. Rogers EM. Diffusion of innovations. Fourth edition ed. New York: Free Press; 1995.

4. Henderson JL, MacKay S, Peterson-Badali M. Closing the research-practice gap: Factors affecting adoption and implementation of a children's mental health program. Journal of Clinical Child and Adolescent Psychology. 2006;35(1):2-12.

5. Proctor EK, Landsverk J, Aarons G, Chambers D, Glisson C, Mittman B. Implementation research in mental health services: an emerging science with conceptual, methodological, and training challenges. Administration and Policy in Mental Health and Mental Health Services Research. 2009;36(1):24-34.
