# Supplementary material for: A type II implementation-effectiveness hybrid quasi-experimental pilot study of a clinical intervention to re-engage people living with HIV into care, ‘Lost & Found’: an implementation science protocol
Source: Pilot Feasibility Stud. 2020 Feb 21;6:29. doi: 10.1186/s40814-020-0559-6 (PMC7035655; doi:10.1186/s40814-020-0559-6)
Supplement: Supplementary file 4 — Additional file 4. DataCollectionTools_CoxLinthwaite [file 40814_2020_559_MOESM4_ESM.docx]

# Additional file 4: Data collection tools

**Table of contents**

[Questionnaire 2](#_Toc11140802)

[Fidelity checklist 8](#_Toc11140804)

[Focus group schedule 11](#_Toc11140807)

# Questionnaire

## **About the questionnaire**

This 38-item self-administered questionnaire is designed to assess acceptability, feasibility, and adoption of the core intervention elements for nurses at the CVIS clinic of the MUHC.

Scales from the TAPP-C (The Arson Prevention Program for Children) Adopter Characteristics Questionnaire and the TAPP-C Innovation Characteristics Questionnaire were adapted to assess adoption [^[[1]](#footnote-2)^]. The TAPP-C questionnaires are comprised of subscales for factors that affect adoption: concern for OOC; self-efficacy; attitude towards the intervention; perceived relative advantage of the intervention; compatibility with existing practices; and complexity of the intervention. Given their conceptual similarities to acceptability and feasibility, the compatibility and complexity subscales were removed to reduce the response burden. Scales for acceptability and feasibility were inspired by Weiner et al.’s pragmatic measures, and the TAPP-C compatibility and complexity subscales [1, ^[[2]](#footnote-3)^].

Acceptability and feasibility are measured separately for each of the core intervention elements, while the adoption subscales focus on the combination of the core elements of the intervention. This was done to reduce response burden for nurses because of the number of items in the TAPP-C questionnaires.

The subscale to which each question belongs is written next to each question in this document and should be removed when being administered (ACC = acceptability, FEAS = feasibility, ADP = adoption, CN = concern, SE = self-efficacy, ATT = attitudes, RA = relative advantage). We also note when questions should be reverse scored.

Importantly, early conceptions of Lost & Found referred to OOC patients as “lost-to-follow-up (LTFU) patients”. This was modified in the protocol for consistency with existing literature, but not modified for questionnaires or CVIS clinical tools, to ensure continuity with previous efforts.

***Scoring:***

Take the mean for all responses within each sub scale (e.g. *ACC, OOC list*) , where “Completely disagree” = 1, “Disagree” = 2, “Neither agree nor disagree” = 3, “Agree” = 4, and “Completely agree” = 5 (for reverse scored questions, it’s still a scale from 1-5 but “Completely disagree” is the highest score).

To calculate the adoption ‘adopter characteristics’ score, take the mean of concern, self-efficacy, and attitude. For the ‘innovation characteristics’ score: (**i**) calculate the mean of the feasibility of the OOC list and feasibility of the phone call; (**ii**) calculate the mean of the acceptability of the OOC list and acceptability of the phone call; then, take the mean of the ‘relative advantage’ subscale, **i** and **ii**.

| Please tick the box that best reflects what you feel about each statement.  ID: ____________________ Date: _____________  DD/MMM/YYYY | | | | | |
| --- | --- | --- | --- | --- | --- |
|  | Completely disagree | Disagree | Neither agree nor disagree | Agree | Completely agree |
| 1. I like the LTFU list. (*ACC, OOC list)* | □ | □ | □ | □ | □ |
| 1. I am happy that we are using the LTFU list (*ACC, OOC list)* | □ | □ | □ | □ | □ |
| 1. The LTFU list is a good fit for our clinic. *(ACC, OOC list)* | □ | □ | □ | □ | □ |
| 1. Using and managing the LTFU list is consistent with the work I usually do. *(ACC, OOC list)* | □ | □ | □ | □ | □ |
| 1. The LTFU list is difficult to use and manage. (*FEAS, OOC list, reverse scored)* | □ | □ | □ | □ | □ |
| 1. It is not possible for me to use or manage the LTFU list. (*FEAS, OOC list, reverse scored)* | □ | □ | □ | □ | □ |
| 1. It takes too much time to coordinate tasks to use and manage the LTFU list. (*FEAS, OOC list, reverse scored)* | □ | □ | □ | □ | □ |
| 1. I like that we are contacting patients who are LTFU. *(ACC, phone calls)* | □ | □ | □ | □ | □ |
| 1. I find it inappropriate that we are contacting patients in their personal time, even though it may be urgent for them to return to care. *(ACC, phone calls, reverse scored)* | □ | □ | □ | □ | □ |
| 1. I am comfortable contacting patients by phone about the importance of ongoing care when they are LTFU. *(ACC, phone calls)* | □ | □ | □ | □ | □ |

ID: ____________________ Date: _____________

DD/MMM/YYYY

|  | Completely disagree | Disagree | Neither agree nor disagree | Agree | Completely agree |
| --- | --- | --- | --- | --- | --- |
| 1. I am happy that we are contacting LTFU patients. *(ACC, phone calls)* | □ | □ | □ | □ | □ |
| 1. Contacting LTFU patients fits well with the clinic’s approach to care. *(ACC, phone calls)* | □ | □ | □ | □ | □ |
| 1. Contacting LTFU patients is consistent with the work I usually do. *(ACC, phone calls)* | □ | □ | □ | □ | □ |
| 1. I always have access to a telephone for contacting patients when I need one. *(FEAS, phone calls)* | □ | □ | □ | □ | □ |
| 1. Communicating by phone is easy. *(FEAS, phone calls)* | □ | □ | □ | □ | □ |
| 1. It takes too much time to coordinate tasks in order to contact patients. *(FEAS, phone calls, reverse scored)* | □ | □ | □ | □ | □ |
| 1. I believe preventing losses to follow up from HIV care is important. *(ADP CONC)* | □ | □ | □ | □ | □ |
| 1. The CVIS should try to re-engage LTFU patients. *(ADP CONC)* | □ | □ | □ | □ | □ |
| 1. I do not think that valuable resources should be directed toward re-engaging LTFU HIV patients. *(ADP CONC, reverse scored)* | □ | □ | □ | □ | □ |
| 1. I don’t understand why losses to follow up are important for the CVIS to address. *(ADP CONC, reverse scored)* | □ | □ | □ | □ | □ |

| ID: ____________________ Date: _____________  DD/MMM/YYYY  **In the remaining questions, we are going to ask you about Lost & Found as a whole.**  ‘Lost & Found’ refers to the combination of 1) using and managing the LTFU list, and 2) contacting patients to re-engage them into care. | | | | | |
| --- | --- | --- | --- | --- | --- |
|  | Completely disagree | Disagree | Neither agree nor disagree | Agree | Completely agree |
| 1. Lost & Found helps me effectively intervene to re-engage LTFU CVIS patients. *(ADP SE)* | □ | □ | □ | □ | □ |
| 1. I can ensure that members of my team are making effective use of available means and resources for contacting patients to re-engage them into care. *(ADP SE)* | □ | □ | □ | □ | □ |
| 1. I am able to effectively collaborate with doctors and allied health workers to address loss to follow up. *(ADP SE)* | □ | □ | □ | □ | □ |
| 1. I am not sure that I can use Lost & Found to effectively re-engage patients. *(ADP SE, reverse scored)* | □ | □ | □ | □ | □ |
| 1. Lost & Found is inadequate for addressing losses to follow up. *(ADP ATT, reverse scored)* | □ | □ | □ | □ | □ |
| 1. I do not believe that Lost & Found improves the quality of care we provide. *(ADP ATT, reverse scored)* | □ | □ | □ | □ | □ |
| 1. Lost & Found improves how our team manages LTFU patients. *(ADP ATT)* | □ | □ | □ | □ | □ |
| 1. Patients are less likely to experience bad health outcomes caused by long absences from care thanks to Lost & Found. *(ADP ATT)* | □ | □ | □ | □ | □ |

|  | Completely disagree | Disagree | Neither agree nor disagree | Agree | Completely agree |
| --- | --- | --- | --- | --- | --- |
| 1. Lost & Found is effective for re-engaging patients into care. *(ADP ATT)* | □ | □ | □ | □ | □ |
| 1. Lost & Found helps me deal more effectively with patient care overall. *(ADP ATT)* | □ | □ | □ | □ | □ |
| 1. Use and management of the LTFU list and phone calls for re-engaging LTFU patients should be used by the CVIS. *(ADP ATT)* | □ | □ | □ | □ | □ |
| 1. I am not in favor of resources being used for Lost & Found. *(ADP ATT, reverse scored)* | □ | □ | □ | □ | □ |
| 1. Lost & Found is a worthwhile initiative. *(ADP ATT)* | □ | □ | □ | □ | □ |
| 1. Lost & Found provides the kinds of tools I like for addressing patient loss to follow up. *(ADP ATT)* | □ | □ | □ | □ | □ |
| 1. I would recommend a similar project to other HIV care centres. *(ADP ATT)* | □ | □ | □ | □ | □ |
| 1. Lost & Found is more effective for re-engaging LTFU patients than what we did before. *(ADP RE)* | □ | □ | □ | □ | □ |
| 1. Lost & Found improves the overall quality of CVIS patient care. *(ADP RE)* | □ | □ | □ | □ | □ |
| 1. Compared to what we did before, Lost & Found is not any better for tracking or re-engaging patients who are LTFU. *(ADP RE, reverse scored)* | □ | □ | □ | □ | □ |

ID: ____________________ Date: _____________

DD/MMM/YYYY

# Fidelity checklist

## **About the fidelity checklist**

This fidelity checklist is designed to assess nurses’ fidelity to core elements and peripheral components of our intervention, which cannot be measured through RISQ (e.g. adherence to or use of motivational communication techniques, RISQ use). These measures were developed specifically for our intervention.

Nurses are expected to communicate with OOC patients using motivational communication techniques. For example, nurses will communicate with OOC patients in a non-judgemental manner, using guiding rather than directive language, as per motivational communication principles [1]. The nurses will use a script (in French or English depending on the patient’s preferred language) as a guide for this conversation, which will include questions regarding barriers to re-engagement that can be addressed over the phone. On a case-by-case basis, nurses will use available clinic resources to assist patients in re-engagement, including connecting them with one of the clinic social workers. Clinic nurses will receive a half-day training session from an expert in motivational communication to build on their existing techniques and clinical experience. In the event of a voicemail, a message will be left for patients that ensures non-disclosure and encourages immediate contact with one of the nurses. Questions to assess adherence to motivational communication were inspired by the Behaviour Change Counselling Scale (BCCS) [^[[3]](#footnote-4)^].

Early conceptions of Lost & Found referred to OOC patients as “lost-to-follow-up (LTFU) patients”. This was modified in the manuscript for consistency with existing literature, but not modified for questionnaires or MUHC clinical tools, to ensure continuity with previous efforts at the clinic.

***Scoring:***

*For questions 1 and 3,* answers are given a score of 1 for ‘Yes’ and 0 for ‘No.’

*For questions 2, 4, and 5,* answers are given a score of 1 for ‘Most of the time’, 2/3 for ‘Some of the time’, 1/3 for ‘Seldom’, and 0 for ‘Never’.

*For question 6,* the first four answers are given a score of 2 for ‘A great deal’, 1 for ‘A little bit’, an 0 for ‘Not at all’. For the last two, answers are given a score of 2 for ‘Yes’ and a score of 1 for ‘No’. Scores for question six are added up for a total score out of a possible twelve.

# Lost & Found Fidelity Checklist

1. **Are you using the LTFU list on the front page of RISQ? □ Yes □ No** Explain (if necessary): ________________________________________

______________________________________________________

1. **Are you using the LTFU list *as intended*?** By ‘*as intended*’ we mean prioritizing patients in the highest risk category with the longest absences (i.e. going through the list from top to bottom)**.**

**□ Never □ Seldom □ Some of the time □ Most of the time** Explain (if necessary): ________________________________________

______________________________________________________

1. **Are you using the RISQ ‘Follow-up’ tab? □ Yes □ No** Explain (if necessary): ________________________________________

______________________________________________________

1. **Are you using the ‘Follow-up’ tab as intended?** By ‘*as intended’* we mean conducting all follow-up or re-engagement tasks in RISQ (i.e. no need for paper or other programs, other than to retrieve information not found in RISQ).

**□ Never □ Seldom □ Some of the time □ Most of the time** Explain (if necessary): ________________________________________

______________________________________________________

1. **When speaking with patients, did you use the same or a similar approach to the one detailed in the re-engagement phone call guide?**

**□ Never □ Seldom □ Some of the time □ Most of the time**Explain (if necessary): ________________________________________

______________________________________________________

1. **Thinking about the calls you’ve made this week, rate the following statements:**

|  | Not at all | A little bit | A great deal |
| --- | --- | --- | --- |
| I made patients feel like they were doing something “bad” or “wrong”. | □ | □ | □ |
| I was able to reflect back (in my own words) the patient’s thoughts and feelings about their situation. | □ | □ | □ |
| I tried to get patients to return to care by giving advice that may have included persuading, guilting, threatening or coercing. | □ | □ | □ |
| I did not ask before providing patients with information or advice. | □ | □ | □ |

| I asked how important patients thought it was to return to care (on a scale from 0 to 10).   □ Yes □ No |
| --- |
| I asked how confident patients felt (on a scale from 0 to 10) in their ability to return to care.  □ Yes □ No |

# Focus group schedule

## **About the focus group schedule:**

These focus group questions are designed for a semi-structured focus group with nurses in order to identify implementation determinants and areas for improvement. Specifically:

- the first question is intended to capture general sentiments about Lost & Found and its implementation as a whole;
- the second question is intended to capture barriers to Lost & Found implementation;
- the third question is intended to capture facilitators to Lost & Found implementation; and
- the fourth question is intended to capture specific changes/adaptations that could be made to improve Lost & Found implementation.

After each question, further probing questions can be asked by the person conducting the focus group in order to obtain additional information or context from participants, where necessary. Questions are worded similarly across implementation phases but catered to the phase in which they are being asked (pre-implementation, implementation, or sustainability phase).

# Focus group interview schedule

| **Pre-implementation**  (One time, prior to implementation) | **Implementation**  (At months 1, 2, 3,6, & 12) | **Sustainability**  (Once, in month 15) |
| --- | --- | --- |
| 1. What do you think about doing Lost & Found? 2. What difficulties do you think you will face? 3. What could make doing Lost & Found easier? 4. How can Lost & Found be improved? | 1. What has it been like doing Lost & Found? 2. What difficulties have you faced? 3. What makes doing Lost & Found easier? 4. How can Lost & Found be improved? | 1. What do you think about the long-term maintenance of Lost & Found? 2. What difficulties do you think you will face with Lost & Found in the future? 3. What about Lost & Found could make its long-term maintenance possible? 4. How can Lost & Found be improved? |

1. . Henderson JL, MacKay S, Peterson-Badali M. Closing the research-practice gap: Factors affecting adoption and implementation of a children's mental health program. *Journal of Clinical Child and Adolescent Psychology*. 2006;35(1):2-12. [↑](#footnote-ref-2)
2. . Weiner BJ, Lewis CC, Stanick C, Powell BJ, Dorsey CN, Clary AS, et al. Psychometric assessment of three newly developed implementation outcome measures. *Implementation Science*. 2017;12(1):108. [↑](#footnote-ref-3)
3. 1. Vallis M. Behaviour Change Counselling—How Do I Know If I Am Doing It Well? The Development of the Behaviour Change Counselling Scale (BCCS). *Canadian journal of diabetes*. 2013;37(1):18-26. [↑](#footnote-ref-4)
