# Supplementary material for: A type II implementation-effectiveness hybrid quasi-experimental pilot study of a clinical intervention to re-engage people living with HIV into care, ‘Lost & Found’: an implementation science protocol
Source: Pilot Feasibility Stud. 2020 Feb 21;6:29. doi: 10.1186/s40814-020-0559-6 (PMC7035655; doi:10.1186/s40814-020-0559-6)
Supplement: Supplementary file 5 — Additional file 5. DataManagement_CoxLinthwaite [file 40814_2020_559_MOESM5_ESM.docx]

# **Additional file 5: Data management**

**Data forms and data entry**

All patient data are collected electronically through routine use of RISQ software. Questionnaires and fidelity checklists will be completed on paper, and responses will be entered into password protected spreadsheets on the MUH servers by the research coordinator. Paper questionnaires and fidelity checklists will be kept in a locked cabinet at the MUHC. Data entry will be double checked for each fidelity checklist and questionnaire.

Raw focus group recordings will be transcribed by one administrative assistant at the MUHC clinic, after which they will be password protected on the MUHC servers. Participant names will be replaced with their study codes on the transcripts. Transcripts will be password protected and kept on the MUHC servers. The two non-nurses present in the focus groups (the research coordinator and a co-investigator who are not CVIS clinical staff) will read and code the transcripts.

Questionnaires and fidelity checklists will to be stored in numerical order and stored in a secure location. Participant files will be maintained in storage for a period of 7 years after completion of the study.

**Data coding**

Audit trails will be maintained for all data coding and analysis in this study. Code for quantitative data import and analysis will be written in R statistical software. Coding for qualitative data will be conducted in Atlas.ti version 8. All codes will be saved on the MUHC servers.
